# Medical Items

**Published:** 1916-01

**Authors:** 


### MEDICAL ITEMS

### ADVANTAGES OF PASADYNE.

Its innocuousness, a quality it possesses notwithstanding its singularly potent therapeutic properties, makes PASADYNE (Daniel) a particular value in women and children or for long continued administration.

